# Isolation and Screening of the Novel Multi-Trait Strains for Future Implications in Phytotechnology

**DOI:** 10.3390/microorganisms13081902

**Published:** 2025-08-15

**Authors:** Zhuldyz Batykova, Valentina Pidlisnyuk, Aida Kistaubayeva, Sergey Ust’ak, Irina Savitskaya, Laila Saidullayeva, Aigerim Mamirova

**Affiliations:** 1 Department of Biotechnology, Faculty of Biology and Biotechnology, Al-Farabi Kazakh National University, Al-Farabi 71, Almaty 050040, Kazakhstan; batyqova@gmail.com (Z.B.); kistaubayeva.kaznu@gmail.com (A.K.); irasava_2006@mail.ru (I.S.); 2 Department of Environmental Chemistry & Technology, Faculty of Environment, Jan Evangelista Purkyně University, Pasteurova 1, 400 96 Ústí nad Labem, Czech Republic; valentyna.pidlisniuk@ujep.cz; 3 Czech Agrifood Research Centre, 430 01 Chomutov, Czech Republic; ustak@vurv.cz; 4 Department of Biotechnology, Auezov South Kazakhstan University, Tauke Khan 5, Shymkent 160012, Kazakhstan; leyla-87.87@mail.ru

**Keywords:** microbial isolates, PGPR inoculants, P and Zn solubilisation, indole-3-acetic acid production, antifungal activity, seed germination

## Abstract

Plant growth-promoting rhizobacteria (PGPRs) colonise the rhizosphere and root surfaces, enhancing crop development through a variety of mechanisms. This study evaluated microbial strains isolated from *Triticum aestivum* L. for key plant growth-promoting traits, including indole-3-acetic acid (IAA) production, phosphate and zinc (Zn) solubilisation, nitrogen (N_2_) fixation, and antifungal activity. Among 36 isolates, 3 (AS8, AS23, AS31) exhibited strong growth-promoting potential. IAA production, citrate assimilation, carbohydrate fermentation, and catalase activity were observed to a comparable extent among the selected strains. AS8 showed the highest protease, lipase, and amylolytic activity, while AS23 demonstrated superior phosphate and Zn solubilisation. Notably, AS31 emerged as the most promising multi-trait isolate, exhibiting the highest levels of IAA production, N_2_ fixation, antifungal activity against five phytopathogens (*Fusarium graminearum*, *F. solani*, *F. oxysporum*, *Pythium aphanidermatum*, and *Alternaria alternata*), potentially linked to its hydrogen sulphide (H_2_S) production, and cellulolytic activity. Molecular identification based on 16S rRNA gene sequencing revealed the isolates as *Stenotrophomonas indicatrix* AS8, *Pantoea agglomerans* AS23, and *Bacillus thuringiensis* AS31. Seed germination assays confirmed the plant growth-promoting efficacy of these PGPR strains, with vigour index increases of up to 43.4-fold. Given their positive impact on seed germination and significant Zn-solubilising abilities, the selected strains represent promising candidates for use as bio-inoculants, offering a sustainable and eco-friendly strategy to enhance agricultural productivity in nutrient-deficient soils. Future research should validate the efficacy of these PGPR strains under pot conditions to confirm their potential for practical agricultural applications.

## 1. Introduction

The global population is projected to reach 9 billion by 2050, placing considerable pressure on food security and necessitating urgent measures to increase crop yields [1] and advocate strategies against hunger and malnutrition [2]. Although the long-term application of chemical fertilisers has traditionally improved agricultural productivity, it has also resulted in detrimental effects on both the environment and human health [3]. Excessive use of synthetic fertilisers contributes to soil degradation through nutrient imbalances, acidification, and depletion of organic matter [4,5]. Currently, Kazakhstan’s vast agricultural territories face significant challenges linked to abovementioned soil degradation [6,7]. In addition, nutrient leaching into groundwater and surface water bodies promotes eutrophication and raises the risk of contamination, posing direct threats to human health. These environmental and health concerns underscore the urgent need for sustainable, bio-based alternatives, such as plant growth-promoting rhizobacteria (PGPRs), to maintain soil fertility, improve crop yields, and promote long-term agricultural sustainability. In this content, the development of locally adapted bio-inoculants derived from native PGPR strains represents a promising strategy to address these constraints by increasing nutrient availability, fostering beneficial microbial interactions, and supporting sustainable agricultural systems tailored to the region’s specific agroecological conditions [6,8]. Furthermore, the abovementioned challenges have catalysed a shift away from conventional intensive agriculture towards alternative, more sustainable practices. As part of this transition, the European Green Deal has set a goal to convert at least 25% of the EU’s agricultural land to organic farming [9], aiming to ensure optimal nutrient supply to crops while simultaneously controlling pathogens and pests through environmentally sound approaches [10,11].

As a component of alternative agricultural practices, PGPRs have attracted considerable interest due to their capacity to enrich soil nutrient content, act as antagonists against various pathogens [12,13], promote plant development, enhance phytoremediation process [14,15,16,17,18], and improve plant tolerance to abiotic stress [17,19,20,21,22]. Although the mechanisms of PGPR-mediated enhancement of plant growth and yield are not yet fully elucidated, their beneficial effects are increasingly recognised, stimulating growing interests in this area of research [18,23,24]. Microbial inoculants can support plant growth directly or indirectly by performing key functions such as nitrogen (N_2_) fixation [25,26], nutrient solubilisation, and mineralisation [27]. Additionally, the secretion of siderophores, antibiotics, enzymes, and phytohormones such as indole-3-acetic acid (IAA), auxins, cytokinins, gibberellins, and ethylene contributes to enhanced plant defence mechanisms [18,23,24,28,29,30].

Zinc (Zn), an essential micronutrient for plant development, plays a vital role in chlorophyll synthesis, enzyme activation in auxin and glucose metabolism, and the biosynthesis of proteins, lipids, and nucleic acids [22,31]. Zn-solubilising bacteria have been shown to significantly increase crop yields [32,33,34], while also enhancing plant resilience by activating defence pathways that improve tolerance to drought, salinity, and extreme temperatures [35]. Similarly, phosphate-solubilising bacteria facilitate phosphorus uptake and contribute to increased yields [24,36]. Isolates obtained from sunflower roots, for instance, improved soil phosphorus availability in response to various environmental stimuli [37,38,39]. Furthermore, PGPR inoculation improved the recovery of wheat and rice from salt stress by stimulating hydrolytic-enzyme activities and efficient rhizosphere colonisation [40,41,42,43,44,45].

Moreover, the literature reports a notable increase in PGPR efficacy when immobilised on sorbents [46,47,48], particularly biochar [49], which provides a nutrient-rich substrate that supports microbial survival. The joint application of biochar and PGPRs has been shown to improve soil nutrient content more effectively than either treatment alone [50,51,52]. For example, co-application of biochar (2% *w*/*w*) with *Paenibacillus polymyxa* and *Bacillus amyloliquefaciens* led to an 87% and 139% increase in soil nitrate and ammonium content, respectively [50]. Jabborova et al. [51] reported significant increases in soil nitrogen (73%), phosphorus (173%), and potassium (17%) following the application of *Bradyrhizobium japonicum* and *Pseudomonas putida* with 3% maize-derived biochar, compared to biochar alone. Similarly, Ren et al. [52] observed that co-application of *Bacillus megaterium* (a N_2_-fixing strain) with wheat-derived biochar resulted in substantial increases in nitrate (by 68% and 22%), inorganic nitrogen (by 45% and 16%), and total potassium (by 21% and 30%) content compared to treatments with either PGPR or biochar alone. The same combination with biochar derived from agricultural waste increased levels of organic carbon (by 16%), available phosphorus (by 79%), and available nitrogen (by 15%) relative to control treatments. Saxena et al. [47] reported that shoot nitrogen concentration reached 1.64 mg N g^−1^ in soil treated with PGPR co-inoculated with biochar, compared to 1.24 and 1.31 mg N g^−1^ in soils treated with PGPR or biochar alone, respectively. Despite the growing body of evidence demonstrating the synergistic benefits of PGPR and biochar, the outcomes remain inconsistent and are largely determined by the specific characteristics of the bacterial isolates and the properties of the biochar used [53,54,55].

The present study aimed to achieve the following: (i) isolate native microbial strains from the rhizosphere of *Triticum aestivum* L. cv. Almaly grown in Almaty region, Kazakhstan; (ii) screen the isolated strains for plant growth-promoting traits, including IAA production, phosphate and Zn solubilisation, N_2_ fixation, and antifungal activity; and (iii) identify the most effective strains based on their impact on the seed germination of *T. aestivum* cv. Almaly.

## 2. Materials and Methods

### 2.1. Soil Collection

Soil samples were collected on 18 March 2023 from the rhizosphere of 8-week-old *Triticum aestivum* L. cv. Almaly (*T. aestivum*) plants grown in a field of the Research Institute of Agriculture and Plant Growing, located in v. Almalybak, Almaty region, Kazakhstan (GPS coordinates: 43°13′46.39″ N 76°41′29.5″ E). The isolation and characterisation of isolated strains were conducted between March 2023 and January 2024.

The soil collection procedure generally followed the ISO 18400-206:2018 [56] standard for on-site operations. A 5 × 5 m plot was selected, from which 4–5 plants were randomly excavated. Immediately after excavation, the aboveground biomass was removed using scissors sterilised with 70% ethanol [57]. The remaining belowground parts (roots) were shaken to remove loosely adhering bulk soil. The roots, along with the attached rhizosphere soil, were then placed into sterile zip-lock bags and transported to the laboratory for further processing [58].

### 2.2. Isolation of Microbial Strains

**Figure 1:**
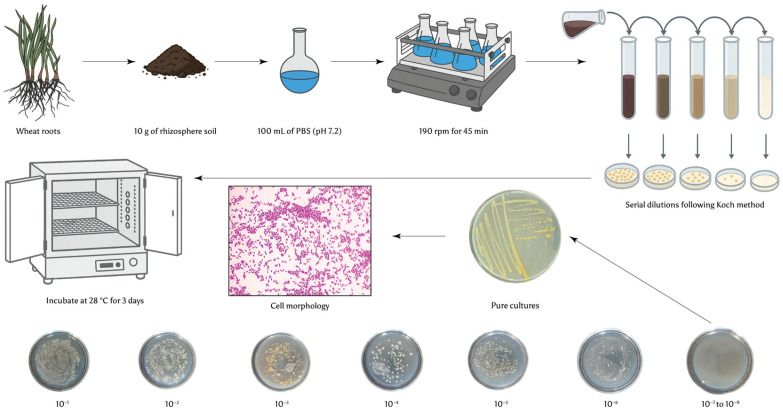
Experimental layout.

Upon arrival at the laboratory, the collected roots were immediately processed to obtain rhizosphere soil, following the procedure described by McPherson et al. [57]. Microbial isolation was performed using the serial dilution method [33], as illustrated in Figure 1. For isolation, 10 g of rhizosphere soil was suspended in 100 mL of sterile phosphate-buffered saline (PBS, pH 7.2) and shaken at 190 rpm for 45 min. The resulting suspension was serially diluted from 10^−1^ to 10^−9^ CFU in autoclaved test tubes containing 9 mL of distilled water (dH_2_O) [59]. From each dilution, 0.1 mL was pipetted and spread onto solid Luria–Bertani (LB) medium (Himedia, India), the composition of which is presented in Table 1. The inoculated plates were incubated at 28 °C for 3 days. Following incubation, well-isolated colonies from the selected dilutions were counted to estimate the number of colony-forming units (CFU) per gram of soil. To maximise the diversity of isolated bacterial strains, 3–5 morphologically distinct colonies were selected from each plate.

Single colonies were isolated and streaked onto sterile LB agar plates to obtain pure cultures. The plates were incubated at 28 °C for 3 days. To ensure culture purity, individual colonies were selected and further purified through repeated subculturing on solid media. Well-isolated colonies were then examined for their morphological characteristics, including colony colour, margins, surface texture, shape, elevation, and pigmentation in accordance with Mohite [60].

### 2.3. Screening Microbial Isolates for Growth-Promoting Properties

#### 2.3.1. Indole-3-Acetic Acid (IAA) Synthesis Assay

The ability of bacterial strains to synthesise IAA was assessed using a tryptophan nutrient broth (TNB), which consisted of LB medium supplemented with 0.5 g of L-tryptophan (Table 1) [61]. A standard calibration curve (Serva, Islandia, NY, USA) was prepared in the range of 10–100 µg mL^−1^ to estimate IAA concentration [62]. Following inoculation, the bacterial strains were incubated in a shaker incubator at 28 ± 2 °C for 3 days. Each strain was tested in triplicate (three tubes per strain). After incubation, the cultures were centrifuged at 6000 rpm for 10–15 min. One (1) mL of the resulting supernatant was mixed with 2 mL of Salkowski’s reagent (prepared by combining 35 mL of 35% HCIO_4_ and 1 mL of 0.5 M FeCl_3_ × 6H_2_O), and the mixture was incubated in the dark for 30 min. The gradual development of a cherry-red colour indicated IAA production [63]. The absorbance of the final solution was measured at 530 nm using a UV–visible spectrophotometer (UV-1900, Shimadzu Corporation, Kyoto, Japan), and the IAA concentration was determined using equation of the calibration curve.

#### 2.3.2. Phosphate Solubilisation Assay

A phosphate solubilisation assay was performed using Pikovskaya medium supplemented with tricalcium phosphate (Ca_3_(PO_4_)_2_) (Table 1) [62]. Both qualitative and quantitative assessments were conducted. Bacterial cultures were spot-inoculated onto the medium in triplicate (three plates per culture) and incubated at 28 ± 2 °C for 6–7 days. The formation of a clear halo zone around the colonies was considered indicative of phosphate-solubilising activity. Following incubation, the phosphate-solubilising potential of each isolate was evaluated by calculating the solubilisation index (*SI*) according to the following equation [64]:

(1) $$SI=\frac{d_{Colony}+d_{Halo\;zone}}{d_{Colony}}$$

where *d*—diameter.

#### 2.3.3. Determination of N_2_ Fixation Activity

Nitrogen (N_2_) fixation was evaluated using Ashby’s N_2_-free medium (Table 1), supplemented with calcium carbonate (CaCO_3_), as a predictive indicator [28]. This medium supports the selective growth of N_2_-fixing bacteria. The isolates were incubated on the medium at 28 ± 2 °C for 7 days in triplicate (three plates per culture). Upon visible colony formation, a single colony was re-streaked onto fresh Ashby’s medium to confirm growth consistency. The appearance of bacterial colonies on N_2_-free medium during incubation was considered indicative of the strain’s N_2_-fixing ability [65].

#### 2.3.4. Determination of Antifungal Activity

Antifungal activity was assessed using the plate–hole diffusion method [66]. Phytopathogenic fungal strains, including *Fusarium graminearum*, *Fusarium solani*, *Fusarium oxysporum*, *Alternaria alternata*, and *Pythium aphanidermatum*, were sourced from the culture collection of the Department of Biotechnology, Al-Farabi Kazakh National University, Kazakhstan.

A 100 μL suspension of fungal spores, containing 10^6^ spores mL^−1^, was inoculated onto the surface of Sabouraud dextrose agar (SDA) medium (Table 1) [44]. Subsequently, 100 μL of a bacterial suspension containing 10^6^ CFU mL^−1^ was added to two of the three wells punched into each plate. The third well, containing only 100 μL of the fungal suspension (10^6^ CFU mL^−1^), served as the negative control. Experiment was performed in triplicate, i.e., three plates per isolate. Antifungal activity was determined by measuring the diameter of the inhibition zones formed around the wells, indicating suppression of fungal growth by the bacterial strains.

#### 2.3.5. Zinc (Zn) Solubilization Assay

The Zn-solubilising potential of the isolated strains was assessed using a modified Bunt and Rovira (B&R) medium (Table 1) [43]. Each strain was spot-inoculated onto B&R medium supplemented with zinc oxide (ZnO) as an insoluble Zn source at a concentration of 1.0 mg mL^−1^ [35] and incubated at 28 °C for 7 days. Experiment was performed in triplicate, i.e., three plates per strain. The diameters of the colonies and surrounding halo zones were measured at 24, 48, and 72 h. Zn solubilisation efficiency was quantified by calculating the *SI* using Equation (1) [64]. The formation of a clear halo zone around the spot culture indicated strain’s ability to solubilise Zn.

### 2.4. Biochemical Properties of Microbial Isolates

#### 2.4.1. Determination of Citrate Assimilation

Citrate assimilation by rhizobacterial strains was assessed using Simmons citrate agar, prepared according to the composition provided in Table 2. The cultures were incubated at 28 ± 2 °C for 24 h. A positive citrate assimilation reaction was indicated by a colour change in the medium from green to blue within 24 h [67]. Following incubation, the medium was examined for this colour shift to confirm citrate utilisation.

#### 2.4.2. Determination of Carbohydrate Fermentation

The carbohydrate fermentation capacity of the bacterial isolates was assessed using Triple Sugar Iron Agar (TSIA) (Table 2) [68]. The strains were incubated at 30 ± 2 °C for 24 h. Experiment was performed in triplicate, i.e., three tubes per isolate. Fermentation was indicated by colour changes in the medium: yellowing of both the slope and the bottom signified fermentation of dextrose, lactose, and sucrose; a red slope with a yellow bottom indicated dextrose-only fermentation. Blackening of the medium signified hydrogen sulphide (H_2_S) production, while splitting or lifting of the medium indicated gas formation by the isolates [68]. The fermentation process was influenced by the pH of the medium.

#### 2.4.3. Catalase Assay

Catalase activity was assessed by transferring a bacterial colony onto a clean glass slide using a sterile nichrome wire loop [68]. A few drops of 3% hydrogen peroxide (H_2_O_2_) were then added to the slide. The immediate formation of oxygen (O_2_) bubbles indicated positive catalase activity, while the absence of bubbling signified a negative reaction [72]. Experiment was conducted in triplicate for each isolate.

#### 2.4.4. Determination of Amylolytic Activity

Amylolytic activity of the bacterial isolates was assessed using starch agar plates, which were incubated at 28 ± 2 °C for 48 h. Following incubation, a potassium iodide (KI) solution was poured over the dishes and immediately drained. The formation of a clear, colourless halo around the bacterial colonies indicated starch hydrolysis and thus the presence of amylolytic activity [69].

#### 2.4.5. Determination of Protease Activity

The proteolytic activity of the bacterial isolates was evaluated using skim milk agar (SMA) (Table 2). The isolates were incubated at 28 ± 2 °C for 48 h. Following incubation, the presence of a clear halo around the bacterial colonies indicated protease production, as evidenced by the degradation of the skim milk substrate [69,70].

#### 2.4.6. Determination of Lipase Activity

Lipase activity was assessed by culturing the bacterial isolates on egg yolk agar (EYA) and incubating them at 30 ± 2 °C for 48 h (Table 2). After incubation, a saturated solution of copper sulphate (CuSO_4_) was poured over the Petri dishes and left to stand for 10–15 min. Excess reagent was then discarded. The appearance of a greenish-blue halo around the colonies indicated positive lipase activity [70].

#### 2.4.7. Determination of Cellulolytic Activity

Cellulolytic activity was evaluated by culturing the bacterial isolates on CMC agar medium (Table 2), with sodium carboxymethyl cellulose serving as the sole carbon source [71]. The plates were incubated at 30 °C for 3 days. After incubation, the plates were flooded with Gram’s iodine solution (prepared by dissolving 2.0 g potassium iodide (KI) and 1.0 g iodine (I) in 300 mL dH_2_O; HiMedia, Mumbai, India) and left to stand for 3–5 min at 30 ± 2 °C [71]. The formation of a clear hydrolysis zone around the colonies indicated cellulose degradation [73].

### 2.5. 16S rRNA Gene Sequencing

Molecular identification of the bacterial isolates was performed through sequencing of the 16S rRNA gene. Genomic DNA was extracted and purified according to the protocol described by Wilson [74]. PCR amplification was carried out using the universal primers 27F (5′-AGAGTTTGATCCTGGCTCAG-3′) and 806R (5′-GGACTACCAGGGTATCTAAT-3′). Sequencing was performed using the BigDye^®^ Terminator v3.1 Cycle Sequencing Kit (Applied Biosystems, Waltham, MA, USA), and the resulting fragments were analysed on an automated 3730xl DNA Analyzer (Applied Biosystems, Foster, CA, USA) [75].

The evolutionary history was inferred using the Maximum Likelihood method based on the Tamura–Nei model [76]. The tree with the highest log likelihood (−1967.51) was selected, and the percentage of replicate trees in which the associated taxa clustered together was shown next to the branches. Initial trees for the heuristic search were generated automatically using the Neighbour-Join and BioNJ algorithms applied to a matrix of pairwise distances estimated using the Tamura–Nei model. The topology with the highest log likelihood was selected.

The phylogenetic tree was drawn to scale, with branch lengths proportional to the number of substitutions per site. The analysis included 22 nucleotide sequences, considering codon positions 1st, 2nd, 3rd, and noncoding. All positions containing gaps and missing data were excluded using the complete deletion option, resulting in a final dataset comprising 529 positions. Evolutionary analyses were conducted using MEGA 11 software (version 11.0.13, 2022) [77].

#### Phylogenetic Identification of Microbial Isolates

The isolates were phylogenetically identified using a modified method described by La Pierre et al. [78]. The 16S rRNA gene was amplified using universal primers fD1 (5′-AGAGTTTGATCCTGGCTCAG-3′) and rD1 (5′–AAGGAGGTGATCCAGCC-3′) [79]. The PCR reaction mixture (25 µL) contained the following: 2.5 μL of 10× Taq polymerase buffer, 2 μL of each primer (10 pM/100 μL), 2.5 μL of 2 mM dNTPs, 2 μL of 25 mM MgCl_2_, 11.7 μL of nuclease-free H_2_O, 0.3 μL of Taq polymerase (5 U μL^−1^), and 2 μL of template DNA (20 ng μL^−1^).

The PCR protocol included an initial denaturation at 95 °C for 5 min, followed by 30 cycles consisting of 60 sec at 94 °C (denaturation), 50 sec at 55 °C (annealing), and 100 sec at 72 °C (extension), with a final extension at 72 °C for 5 min. Amplified products were resolved by electrophoresis on a 1% agarose gel and visualised under UV light using a gelLITE Gel Documentation System (Cleaver Scientific Ltd., Rugby, UK). Amplicons were purified using a GeneJET PCR Purification Kit (K0701; Thermo Fisher Scientific, Waltham, MA, USA) and sequenced by Sanger method through the services of Macrogen Inc. (Seoul, Republic of Korea). Sequence contigs were constructed by assembling forward and reverse reads.

Gene sequences were compared to publicly available sequences using the NCBI BLAST (version 2.15.0) tool (https://blast.ncbi.nlm.nih.gov/, accessed on 10 June 2025) [80,81]. Closely related sequences were retrieved from public databases, and pairwise comparisons were performed using the Sequence Demarcation Tool v.1.2 [82,83]. Phylogenetic relationships were inferred using the Maximum Likelihood method in MEGA 11, with 1000 bootstrap replicates, facilitated by Macrogen Inc. (Seoul, Republic of Korea).

### 2.6. Seed Germination Assay

The effect of selected PGPR strains on the germination of wheat seeds (*Triticum aestivum* L. cv. Almaly 2023) was evaluated under controlled conditions. The experimental treatments included the following: a non-inoculated control, and inoculation with isolates AS8, AS23, and AS31 (four treatments in total). Each treatment was performed in triplicate (10 seeds per replicate; 30 seeds per treatment; 120 seeds in total). Wheat seeds were surface-sterilised with 0.1% mercuric chloride (HgCl_2_) for 1 min [84] and rinsed four times with sterile dH_2_O. The sterilised seeds were then soaked for 1 h in a bacterial suspension of each isolate under aseptic conditions to ensure uniform surface colonisation. For control treatment, seeds were soaked in sterile dH_2_O. Ten (10) seeds per replicate were placed on sterile filter paper in Petri dishes and incubated at 25 °C for 6 days. Sterile dH_2_O was added daily to maintain moisture at 60–70% [85]. Seedling data were collected on the 14th day after sowing and included measurements of root and shoot lengths. The seedling vigour index (*VI*) was calculated using the following equation [43,44]:

(2) $$VI=\left(RL+SL\right)\times Germination\;rate$$

where *RL*—root length, *SL*—shoot length.

### 2.7. Statistical Analysis

The data analysis was conducted using RStudio software (version 2023.06.0 Build 421, RStudio PBC, 2023). All experimental assays were performed in triplicate. Tukey HSD tests were performed for the pairwise comparisons of the means, while ANOVA was used to confirm statistical significance. In case the Shapiro–Wilk test failed, non-parametric Kruskal–Wallis’s test was applied to determine the significant difference, followed by pairwise comparison with ‘Bonferroni’ adjustment. Subsequently, the treatments were categorised by letter in descending order, and graphs were generated. Significance was declared at *p* < 0.05.

## 3. Results

### 3.1. Isolation and Morphological Characterisation of Microbial Strains

**Figure 2:**
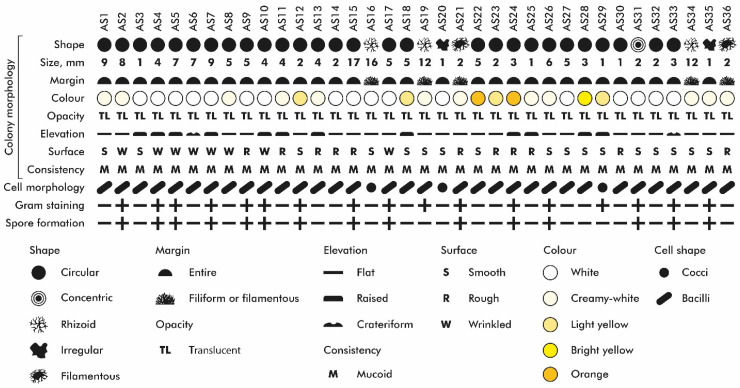
Morphological characterisation of isolated strains.

A total of 36 bacterial isolates were obtained from *T. aestivum* rhizosphere. From all isolated microorganisms, 19 were identified as Gram-negative and 17 as Gram-positive. The collection included 21 non-spore-forming and 15 spore-forming bacteria. Microscopic analysis confirmed 3 isolates with cocci morphology, with the remaining identified as bacilli (Figure 2 and Figure S1).

### 3.2. Screening Microbial Isolates for Plant Growth-Promoting Properties

At the initial stage, a group of promising isolates which demonstrated significant PGP activity was selected. To ascertain their potential, studied isolates were tested for the ability to synthesise the phytohormone IAA, solubilize phosphates, and fix N_2_.

#### 3.2.1. Indole-3-Acetic Acid Production

**Figure 3:**
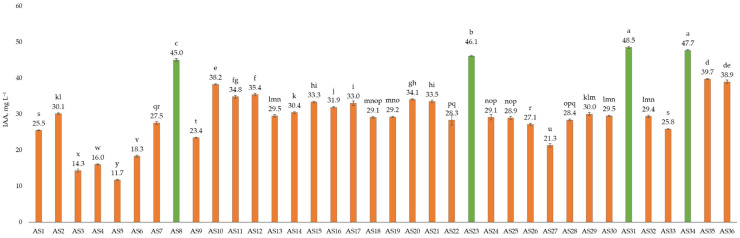
Indole-3-acetic acid production by 36 isolates (*n* = 6). Notes: green-coloured bars indicate the most promising strains with the highest activity; orange-coloured bars present strains with lower activity. Different letters above bars indicate significant difference between isolates’ IAA production ability.

Figure 3 and Figure S2 illustrate the IAA production profiles of 36 bacterial isolates, as a crucial aspect of their recognition as PGPR. Figure 3 shows the production levels of IAA across the different isolates as IAA concentration ranges from 11.7 to 48.5 mg L^−1^, consequently. The isolates AS8, AS23, AS31, and AS34 (green bars) stand out for exceptional IAA production, reaching levels above 45.0 mg L^−1^, with AS31 demonstrated the highest value of IAA production (48.5 mg L^−1^), closely followed in this characteristic by AS34 (47.7 mg L^−1^), AS23 (46.1 mg L^−1^), and AS8 (45.0 mg L^−1^). Conversely, isolate AS5 demonstrated the lowest level of IAA production (11.7 mg L^−1^). These results underscore the potential of specific PGPR isolates to significantly enhance plant growth through hormonal modulation.

The isolates, which dominate the middle range (orange-coloured bars) showed a broad consistency in the value of IAA production, indicating their robust potential for IAA synthesis under the tested conditions (Figure 3). Meanwhile, the isolates, marked in green colour, which surpassed beyond the others, possessed unique metabolic capabilities or genetic traits that allowed them to come through the typical IAA production rates.

#### 3.2.2. Phosphate Solubilisation

**Figure 4:**
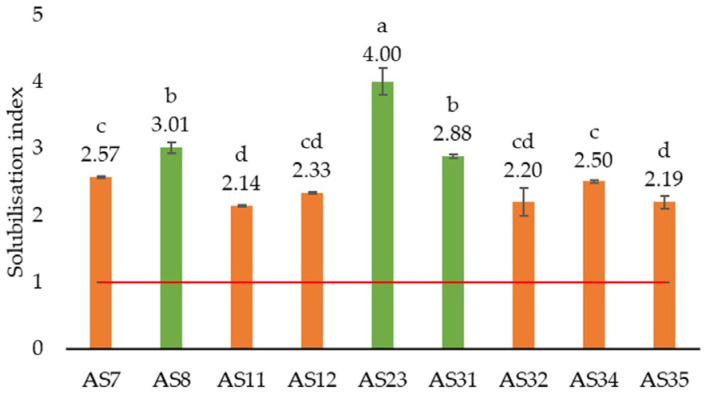
Phosphate solubilization indices on the 7th day of incubation (*n* = 3). Notes: green-coloured bars indicate the most promising strains with the highest value of *SI*; orange-coloured bars present strains with a lower value of *SI*; red line indicate minimal *SI* value showing the presence of solubilization activity. Different letters above bars indicate significant difference between isolates’ phosphate solubilization activity.

Taking into account that *SI* equal to or greater than 1.5 indicates a significant solubilization activity [84], it can be concluded that among 36 isolates obtained, only 9 (AS7, AS8, AS11, AS12, AS23, AS31, AS32, AS34, and AS35) demonstrated the ability to solubilize phosphate (Figure S3). Furthermore, three isolates (AS8, AS23, and AS31) exhibiting the highest solubilization activity with *SI* values ranging from 2.88 to 4.00 were selected for the following investigation (Figure 4).

#### 3.2.3. Nitrogen Fixation

**Figure 5:**
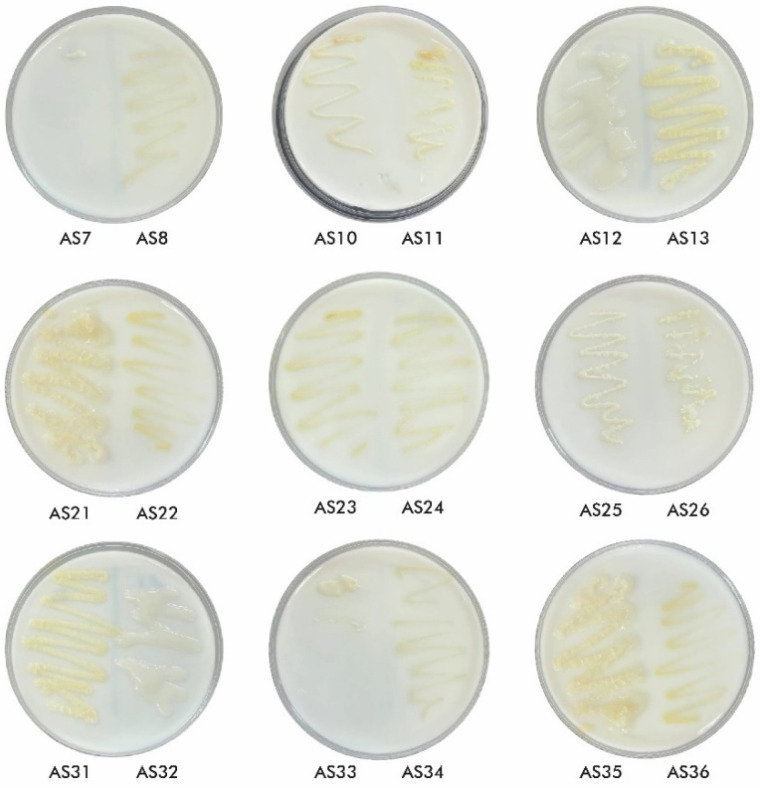
Nitrogen fixation activity of studied isolates. Note: Only isolates capable of N_2_ fixation are shown.

Among 36 studied isolates, only 18 showed the ability to fix N_2_. However, AS7 and AS33 displayed a weak ability to fix N_2_, being characterised by poor growth over 7 days without further development in a zigzag pattern. Conversely, the other 16 isolates effectively fixed N_2_, as evidenced by their pronounced growth (Figure 5).

**Figure 6:**
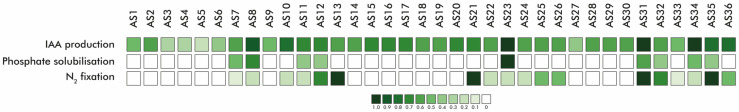
Visual representation of the activity intensity of the tested isolates for three plant growth-promoting traits. Note: Activity values were normalised to a scale from 0 to 1 for heatmap visualisation, where 0 indicates no activity and 1 represents the maximum observed intensity.

Based on the evaluation of three PGP traits (Figure 6), four isolates (AS8, AS23, AS31, and AS34) were initially identified as high performers in terms of IAA production. Subsequent screening for phosphate solubilisation revealed three isolates (AS8, AS23, and AS31) as the best performing candidates. Although isolates AS12, AS13, AS21, AS32, AS35, and AS36 exhibited notable growth on nitrogen-free medium, they were excluded from further analysis due to the predictive, rather than confirmatory, nature of the method used. Therefore, all subsequent analyses focused exclusively on the top three isolates: AS8, AS23, and AS31.

#### 3.2.4. Antifungal Activity

**Figure 7:**
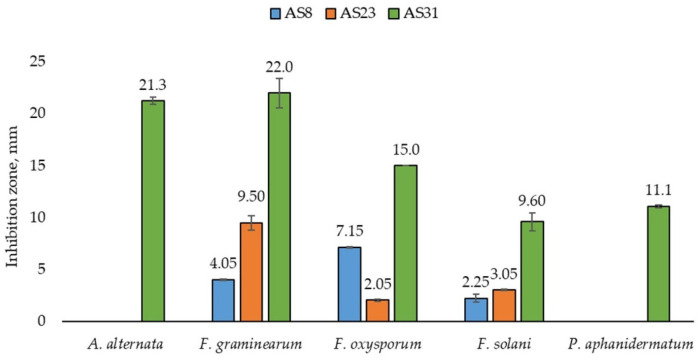
Antifungal activity of the selected isolates.

The studied isolates were tested against five pathogenic fungi: *F. graminearum*, *F. solani*, *F. oxysporum*, *A. alternata*, and *P. aphanidermatum.* As seen from Figure 7, isolates AS8 and AS23 exhibited the antagonism against *F. graminearum*, *F. solani*, and *F. oxysporum*. Isolate AS31 displayed the most significant antifungal activity, antagonising all five tested phytopathogens (Figure 7 and Figure S4).

The inhibition zones measured were as follows: for *F. graminearum*—4.05 ± 0.07 mm by AS8, 9.50 ± 0.71 mm by AS23, and 22.0 ± 1.41 mm by AS31; for *F. solani*—2.25 ± 0.35 mm by AS8, 3.05 ± 0.07 mm by AS23, and 9.60 ± 0.85 mm by AS31; and for *F. oxysporum*—7.15 ± 0.07 mm by AS8, 2.05 ± 0.07 mm by AS23, and 15.0 ± 0 mm by AS31. Notably, only AS31 demonstrated antagonistic activity against *A. alternata* and *P. aphanidermatum*, with growth inhibition zones of 21.3 ± 0.35 mm and 11.1 ± 0.14 mm, respectively (Figure 7).

#### 3.2.5. Zinc Solubilization

**Figure 8:**
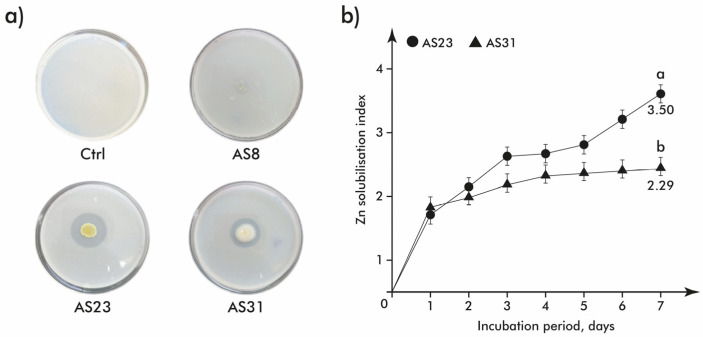
Zn solubilisation by the selected isolates: (**a**) visual representation of inhibition zones; (**b**) *SI* dynamic during incubation (*n* = 3). Different letters indicate significant difference between isolates’ ability to solubilize Zn.

For 3 selected isolates (AS8, AS23, and AS31), the Zn solubilization ability was assessed by observing distinct halo zones around the spot cultures. The results revealed that isolate AS8 did not solubilize ZnO, whereas isolates AS23 and AS31 on the 7th day of incubation achieved *SI* values equal to 3.60 and 2.47, respectively (Figure 8).

### 3.3. Biochemical Characterisation of Promising Strains

All tested isolates exhibited catalase and amylolytic activities and showed the ability to assimilate citrate and ferment carbohydrates (Table 3, Figure S5). Only isolate AS31 demonstrated the ability to produce H_2_S, evidenced by the blackening of the medium. Furthermore, AS8 exhibited protease activity; both microorganisms AS8 and AS31 displayed cellulase and lipase activities, contributing to the degradation of phytopathogens by enzymatically breaking down their cell walls.

Figure S5 illustrates amylase activity of three selected strains, which resulted in formation of the colourless zones around the bacterial colonies. The amylase activity of isolates AS8 and AS31 was notably higher compared to AS23. Both AS8 and AS31 exhibited lipase and protease activities which confirmed by turbidity around the colonies (Figure S5). The cellulase activity was observed in AS8 and AS31 isolates (Figure S5). Considering carbohydrates fermentation, a red slope with a yellow bottom, as observed in strains AS8 and AS23, indicated that only dextrose was fermented (Figure S5). Blackening of the medium and rising, as seen in the test tube with AS31, indicated the isolate’s ability to produce H_2_S and gas (Figure S5).

**Figure 9:**
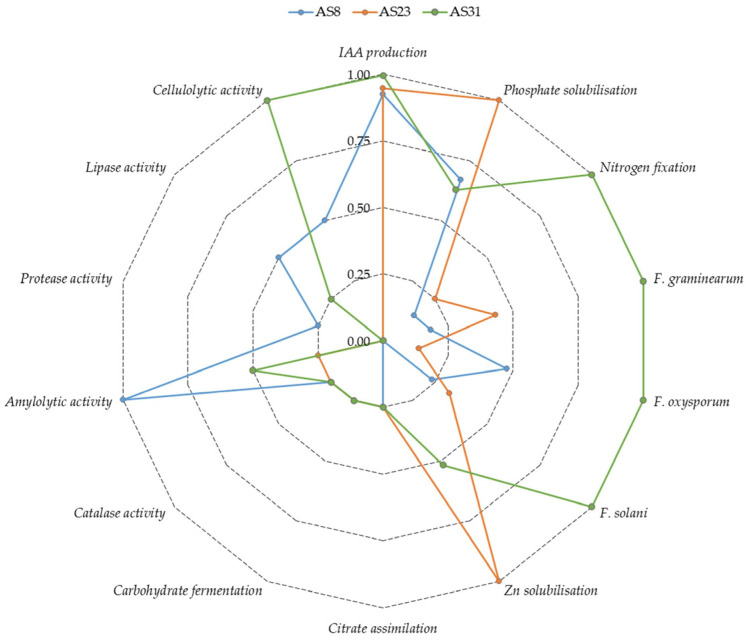
Visual representation of the intensity of functional traits in the selected isolates. Note: Activity values were normalised to a scale from 0 to 1 for heatmap visualisation, where 0 indicates no activity and 1 represents the maximum observed intensity.

A comparative assessment of the three selected isolates revealed the following: (i) AS8 exhibited strong performance in IAA production and phosphate solubilisation, and showed the highest amylolytic activity; (ii) AS23 displayed the greatest activity in both phosphate and Zn solubilisation, with IAA production comparable to that of AS8; and (iii) AS31 demonstrated the highest levels of IAA production, N_2_ fixation, antifungal activity against five phytopathogens, and cellulolytic activity (Figure 9).

### 3.4. Genetic Identification of Promising Strains

**Figure 10:**
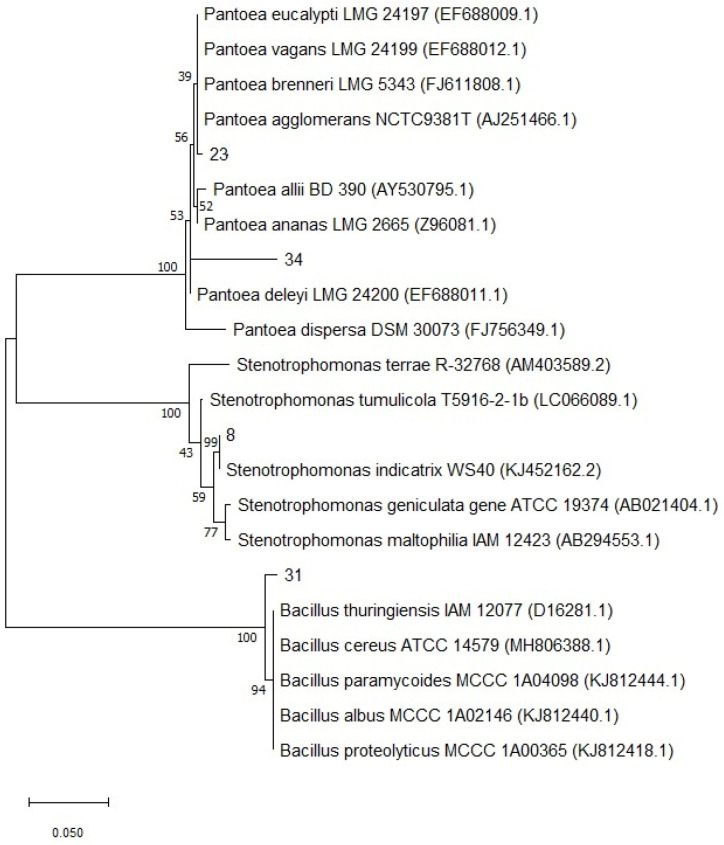
The phylogenetic tree of 16S RNA sequencing.

The analysis of the 16S rRNA gene sequence and the phylogenetic tree indicated that AS8 closely matches *Stenotropomonas indicatrix* (100% affinity), AS23 matches *Pantoea agglomerans* (98.7% affinity), and AS31 matches *Bacillus thuringiensis* (98.5% affinity) (Figure 10). The 16S rRNA gene sequences of these selected bacterial strains were deposited in the GenBank database (NCBI) under the following accession numbers: *S. indicatrix* LMG 29,892 strain-MG273677.1, *B. thuringiensis* strain T32-ON329202.1, and *P. agglomerans* strain HX-1-MK350312.1.

### 3.5. Influence of PGPRs on Seed Germination

The inoculation of wheat seeds with selected PGPR strains significantly influenced the germination rate (Figure S6, Table 4). In particular, seeds inoculated with AS23 demonstrated the most considerable growth, with seedlings reaching up to 6.2 cm in length. The most significant impact on the root development was observed in seeds treated with AS31.

Figure S6 depicts wheat seeds cultivation in Petri dishes under the sterile conditions on day 14. The growth of roots and seedlings of crops inoculated with bacterial strains showed the superior performance compared to the control. On the 14th day, the most significant increase in length was observed for seeds treated with AS31, achieving growth estimated up to 16.6 cm. This isolate also demonstrated the greatest root elongation.

## 4. Discussion

Nowadays, global agriculture faces significant challenges, including water scarcity, soil degradation, climate change, and urbanisation, all of which increasingly exacerbate sustainable food production. Abiotic stress impacts crop performance depending on stress duration and severity, growth conditions, plant genotype [86,87], and environmental factors [88,89]. These processes are additionally compounded by photosynthetic and respiratory activity variations and stress-responsive gene expression [90,91,92]. To address these issues, it is necessary to develop eco-friendly agricultural approaches, such as PGPRs, which were proven to enhance crop productivity and mitigate abiotic stress [93,94].

### 4.1. Plant Growth-Promoting Properties of Isolates

#### 4.1.1. Indole-3-Acetic Acid Production

Indole-3-acetic acid plays a crucial role in root development and abiotic stress mitigation [95]. In this study, the isolates demonstrated substantial IAA production, with *B. thuringiensis* AS31 producing 48.5 mg L^−1^, *P. agglomerans* AS23 producing 46.1 mg L^−1^, and *S. indicatrix* AS8 producing 45.0 mg L^−1^. These results align with earlier findings highlighting the importance of IAA-producing bacteria in enhancing plant growth and stress tolerance [96,97,98,99,100,101]. Moreover, several studies investigated different approaches to optimise IAA production by *Bacillus* sp. and *P. agglomerans* strains through medium supplementation with sucrose, glucose, or maltose [102,103,104,105,106]. Genome analyses of *P. agglomerans* revealed gene clusters (including *ipdC* and *IaaH* genes), participating in key pathways for IAA synthesis, thereby emphasising its role in supporting plant development [40].

#### 4.1.2. Nutrient Solubilization

For nutrient bioavailability, photosynthesis, and cell division phosphate and Zn solubilization are essential traits [107]. Investigated isolates exhibited significant solubilization indices, consistent with studies on wheat rhizosphere bacteria [108,109,110,111,112]. Indeed, *P. agglomerans* was shown to contain *gcd* gene, crucial for phosphate solubilization through glucose dehydrogenase [41,42]. Zn-solubilizing bacteria, such as the isolates received in the current study, produce organic acids like gluconic acid, which lower soil pH and dissolve nutrients, enhancing their availability for plant uptake. These traits are critical for addressing nutrient deficiencies, particularly in cereals [113,114,115], and improving crop yield and Zn biofortification [116,117].

### 4.2. Synergy of Plant Growth-Promoting Traits

The combined action of IAA production, phosphate solubilization, and Zn solubilization significantly enhance plant nutrient uptake. IAA-producing bacteria improve root development, facilitating access to solubilized nutrients (phosphates and Zn). Phosphate-solubilizing bacteria release organic acids that simultaneously make phosphorus and Zn phytoavailable [118,119], while Zn-solubilizing bacteria secrete chelating agents to prevent nutrient precipitation [120,121]. In addition, IAA production often coincides with improvement in phosphate solubilization. Certain PGPRs can increase IAA levels and phosphate solubilization, suggesting that IAA might play a role in modulating microbial gene expression linked to phosphorus metabolism [119,122]. Furthermore, coupling the above traits with N_2_ fixation ability might contribute to enhanced efficiency of microbial strains in supporting plant growth in nutrient-deficient soils [123,124].

### 4.3. Biocontrol Potential of Isolates

#### 4.3.1. Hydrolytic Enzymes Activity

Hydrolytic enzymes such as cellulases, proteases, and lipases play a pivotal role in plant growth promotion and biocontrol mechanisms by targeting structural components of pathogen cell walls. This dual action suppresses pathogen proliferation while improving plant health, contributing to sustainable agriculture [125]. Among studied strains, *S. indicatrix* AS8 demonstrated the activity considering all three enzymes, whereas *P. agglomerans* AS23 did not show any activity.

Cellulases decompose cellulose, a major constituent of plant cell walls and soil organic matter, releasing glucose and other sugars that enrich the soil nutrient profile and foster plant growth [126]. Consequently, cellulases can hydrolyse the cellulose in fungal cell walls, effectively disrupting pathogens and mitigating their proliferation [125]. Thus, being the first course of action, greater cellulase activity of *B. thuringiensis* AS31 could potentially explain its resistance to broader range of fungal pathogens.

Proteases hydrolyse proteins into amino acids and peptides, aiding soil nutrient cycling while also disrupting pathogen protein structures, impairing their functionality and viability [127]. This enhances plant defence mechanisms and suppresses soil-borne diseases, supporting plant growth [128,129]. Since only *S. indicatrix AS8* demonstrated the proteolytic activity, this strain could be considered more beneficial for application in diverse environments.

Lipases catalyse lipid breakdown into fatty acids and glycerol, indirectly benefiting plant health by improving soil fertility and structure via increased decomposition of organic matter and nutrient cycling [130]. While their direct biocontrol role is less pronounced compared to cellulases and proteases, lipases contribute to pathogen suppression by hydrolysing lipid components in cell membranes, causing structural damage and cell lysis [131,132]. Considering indirect action of this enzyme in supporting plant development, PGPR strains (*S. indicatrix* AS8 and *B. thuringiensis* AS31) demonstrating lipase activity might be useful in long-term applications as microbe–sorbent complexes due to potential enhancement of nutrient release from sorbent into the soil.

The synergistic activity of cellulases, proteases, and lipases enhance nutrient availability and pathogen suppression, promoting plant growth and resilience against biotic stress. Understanding these enzymes’ interactions is essential for optimising biocontrol strategies and advancing sustainable agriculture [125].

#### 4.3.2. Antifungal Activity

The isolates exhibited strong antifungal activity, with *B. thuringiensis AS31* showing substantial biocontrol potential against *F. graminearum*, *F. solani*, *F. oxysporum*, *A. alternata*, and *P. aphanidermatum*, whereas *S. indicatrix* AS8 and *P. agglomerans* AS23 demonstrated inhibition potential only against *F. graminearum*, *F. solani*, and *F. oxysporum*. These activities could be attributed to hydrolytic enzymes such as cellulases and proteases [133,134,135,136], which might degrade fungal cell walls, and microbial compounds like agglomerin and pantocin [30]. Indeed, protease and cellulase activity in *S. indicatrix* AS8 and *B. thuringiensis* AS31 could explain their greater antifungal performance, being consistent with findings of Adeleke et al. [137] reporting the production of amylase, cellulase, and protease by *S. indicatrix* BOVIS40 and *B. thuringiensis* BSA123. Meanwhile, *Pantoea* sp. was found to produce antimicrobial compounds such as agglomerin, phenazine, herbicolin, and pantocin, usually utilised against diverse plant diseases [30,97]. Thus, biocontrol potential of studied strains could contribute not only to plant protection from pathogens but also to its overall enzymatic defence system [45,133,134].

### 4.4. Comparative Analysis

**Figure 11:**
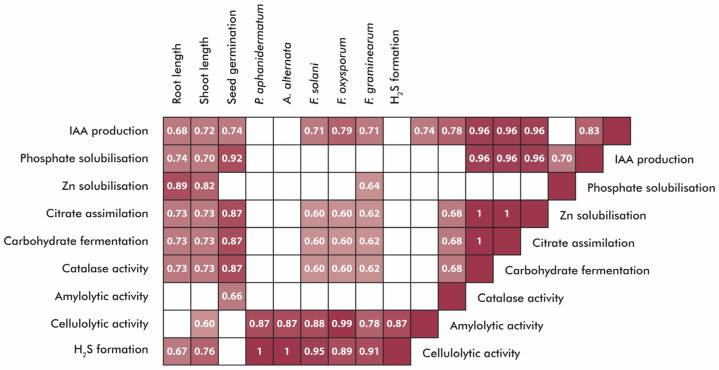
Pearson correlation heatmap. Note: Only significant coefficients are shown.

To evaluate the relationship between plant growth-promoting traits of the selected strains and their influence on seed germination, a Pearson correlation heatmap was generated (Figure 11). N_2_ fixation results were excluded from the analysis due to the predictive nature of the method employed. The Pearson correlation analysis revealed 26 very strong correlations (*r* ≥ 0.9) and 34 strong correlations (0.7 ≤ *r* < 0.9).

Before discussing individual correlations in detail, it is noteworthy that *B. thuringiensis* AS31 exhibited the highest overall microbial activity, particularly in antifungal properties. *S. indicatrix* AS8 showed strong enzymatic activity, while *P. agglomerans* AS23 demonstrated moderate but consistent biochemical performance.

As expected, positive correlations were observed between IAA production, phosphate solubilisation, and seed germination parameters. However, several unexpected yet noteworthy correlations were identified. Perfect correlations (*r* = 1.0) were observed between antifungal activity against *P. aphanidermatum* and *A. alternata* and H_2_S production, as well as among the citrate–carbohydrate–catalase enzymatic complex. One of the most biologically meaningful findings was the near-perfect correlation between resistance to *F. oxysporum* and cellulolytic enzyme activity (*r* = 0.99), which may suggest novel interaction pathways worth further investigation.

Plant growth parameters also exhibited exceptionally strong correlations, with shoot and root lengths showing near-perfect alignment. Overall, the correlation matrix indicated that *B. thuringiensis* AS31 was the principal driver of many of the strongest correlations, particularly in relation to antifungal activity.

## 5. Conclusions

Current study focused on the isolation of microbial strains from the rhizosphere of 8-week-old *T. aestivum* plants cultivated in the field at Almalybak village, Kazakhstan, resulting in the identification of 36 distinct isolates. Among these, three strains (*Stenotropomonas indicatrix* AS8, *Pantoea agglomerans* AS23, and *Bacillus thuringiensis* AS31) demonstrated superior plant growth-promoting traits, including IAA production, phosphate and Zn solubilisation, N_2_ fixation, biocontrol potential, citrate assimilation, carbohydrate fermentation, and enzymatic activity. In vivo germination assays with wheat seeds confirmed the beneficial effects of these PGPR strains, particularly *P. agglomerans* AS23 and *B. thuringiensis* AS31, which significantly enhanced both aboveground and root biomass. This study, integrating both in vitro and in vivo approaches, highlights the potential of the selected PGPRs as eco-friendly bio-inoculants to improve crop productivity and stress resilience.

Acknowledging the inherent limitations of lab-scale studies, future research should focus on validating the efficacy of these strains under pot and greenhouse conditions to ensure scalability and field relevance. Overall, this work represents a foundational step in a broader research framework aimed at developing efficient PGPR–sorbent complexes for sustainable agricultural and environmental applications. The next phase of research will involve the immobilisation of microbial cells onto carrier surfaces, enabling enhanced microbial survival and sustained functional activity. Ultimately, the goal is to improve crop performance in nutrient-deficient agricultural soils and to support remediation of marginal lands, using selected strains as monocultures, consortia, or microbe–sorbent complexes under ex situ conditions.

## Figures and Tables

**Table 1:** The composition of culture media used to assess the growth-promoting properties of isolates.

Component	Unit	LB	TNB	Pikovskaya	B&R	Ashby’s	SDA
Ref.	−	[60]	[61]	[36]	[43]	[28]	[44]
Agarose	g	20	−	−	15	−	15.0
Glucose	g	−	−	10	10	−	40.0
Peptone	g	−	−	−	1.0	−	10.0
Sucrose	g	−	−	−	−	20	−
Tryptone	g	10	10	−	−	−	−
Yeast extract	g	5	5	0.5	1.0	−	−
CaCl_2_	g	−	−	−	−	0.02	−
CaCO_3_	g	−	−	−	−	5	−
Ca_3_(PO_4_)_2_	g	−	−	5	−	−	−
FeCl_3_	g	−	−	−	−	0.01	−
FeSO_4_ ×·7H_2_O	mg	−	−	0.1	−	−	−
KCl	g	−	−	0.2	−	−	−
KH_2_PO_4_	g	−	−	−	−	0.4	−
K_2_HPO_4_	g	−	−	−	0.18	0.1	−
L-tryptophan	g	−	0.5	−	−	−	−
MgCl_2_	g	−	−	−	0.2	−	−
MgSO_4_ ×·7H_2_O	g	−	−	0.1	−	0.2	−
MnSO_4_ ×·7H_2_O	mg	−	−	0.1	−	−	−
NaCl	g	5	0.5	0.2	−	0.1	−
Na_2_MoO_4_	g	−	−	−	−	0.002	−
(NH_4_)_2_SO_4_	g	−	−	0.5	0.5	−	−
dH_2_O	L	1	1	1	1	1	1
pH	−	7.0	7.0	7.0	7.2	6.9	5.6

Notes: LB—Luria–Bertani medium; TNB—tryptophan nutrient broth medium; B&R—Bunt and Rovira medium; SDA—Sabouraud dextrose agar.

**Table 2:** The composition of culture media used for the determination of isolates’ biochemical properties.

Component	Unit	Simmons	TSIA	SMA	EYA	CMC
Ref.	−	[67]	[68]	[69,70]	[70]	[71]
Agarose	g	15.0	12.0	15.0	25.0	20.0
Beef extract	g	−	3.0	−	−	−
Bromo thymol blue	g	0.08	−	−	−	−
Casein	g	−	−	5.0	−	−
CMC	g	−	−	−	−	0.2
Dextrose	g	−	1.0	−	−	−
Glucose	g	−	−	1.0	−	2.0
Lactose	g	−	10.0	−	−	−
Peptone	g	−	20	−	−	1.0
Phenol red	g	−	0.024	−	−	−
Proteose peptone	g	−	−	−	40.0	−
Skim milk	percent	−	−	7.0	−	−
Sucrose	g	−	10.0	−	−	−
Yeast extract	g	−	3.0	2.5	−	−
C_34_H_32_ClFeN_4_O_4_	g	−	−	−	0.005	−
FeSO_4_ ×·7H_2_O	g	−	0.01	−	−	0.01
KCl	g	−	−	−	−	0.3
KH_2_PO_4_	g	−	−	−	1.0	1.0
K_2_HPO_4_	g	1.0	−	−	−	−
MgSO_4_ ×·7H_2_O	g	0.2	−	−	0.1	0.3
NaCl	g	5.0	2.0	−	2.0	0.1
dH_2_O	L	1	1	1	1	1
pH	**−**	7.0	7.0	7.0	7.2	6.9

Notes: TSIA—triple sugar-iron-agar medium; SMA—skim milk-agar medium; EYA—egg yolk-agar medium; CMC—carboxymethyl cellulose.

**Table 3:** Biochemical properties of the selected isolates.

Parameter	AS8	AS23	AS31
Citrate assimilation	+	+	+
Carbohydrate fermentation	+	+	+
Catalase activity	+	+	+
Amylolytic activity	+++	+	++
Protease activity	+	−	−
Lipase activity	++	−	+
Cellulolytic activity	++	−	+++

Notes: +++ strong activity; ++ moderate activity; + weak activity; − absence of activity.

**Table 4:** Seed germination influenced by selected isolates (*n* = 3). Different letters indicate significant difference between treatments within one parameter.

Treatment	*SL*, cm	% to Ctrl	*RL*, cm	% to Ctrl	*VI*	% to Ctrl
Day 7						
Ctrl	0.40 ± 0.14 b	100	−	100	24.0 ± 8.49 b	100
AS8	0.50 ± 0.08 b	−	0.18 ± 0.10 b	−	45.0 ± 10.4 b	−
AS23	4.22 ± 0.67 a	1 055	1.12 ± 0.26 a	−	558 ± 45.5 a	2 325
AS31	5.25 ± 0.17 a	1 313	1.38 ± 0.17 a	−	580 ± 51.2 a	2 415
*p*-value	<0.001		<0.01		<0.001	
Day 14						
Ctrl	3.40 ± 1.27 d	100	0.46 ± 0.11 c	100	40.0 ± 6.53 d	100
AS8	6.78 ± 0.36 c	199	1.04 ± 0.18 b	227	784 ± 66.6 c	1 960
AS23	12.7 ± 0.72 b	374	2.46 ± 0.33 a	534	1 540 ± 105 b	3 850
AS31	16.6 ± 0.73 a	488	2.83 ± 0.29 a	616	1 737 ± 70.2 a	4 343
*p*-value	<0.001		<0.001		<0.001	

Notes: *SL*—shoot length; *RL*—root length; *VI*—vigour index.

## Data Availability

The original contributions presented in this study are included in the article/Supplementary Materials. Further inquiries can be directed to the corresponding author.

## Supplementary Materials

The following supporting information can be downloaded at: https://www.mdpi.com/article/10.3390/microorganisms13081902/s1. Figure S1: Microscopic analysis of isolated microbial strains; Figure S2: Qualitative analysis of IAA production by isolated microbial strains; Figure S3: Qualitative analysis of phosphates solubilisation by isolated microbial strains; Figure S4: Antifungal activity of selected isolates; Figure S5: Biochemical properties of selected isolates: (a) carbohydrates fermentation; (b) amylolytic activity; (c) protease activity; (d) lipase activity; (e) cellulolytic activity; Figure S6: Influence of selected isolates on *Triticum aestivum* seed germination.

## Abbreviations

The following abbreviations are used in this manuscript:

PGPR	Plant growth-promoting rhizobacteria
PGP	Plant growth-promoting
IAA	Indole-3-acetic acid
