# Supplementary material for: Isolation and Screening of the Novel Multi-Trait Strains for Future Implications in Phytotechnology
Source: Microorganisms. 2025 Aug 15;13(8):1902. doi: 10.3390/microorganisms13081902 (PMC12388201; doi:10.3390/microorganisms13081902)
Supplement: Supplementary file 1 [file microorganisms-13-01902-s001.zip › microorganisms-3724376-supplementary.pdf]

Supplementary materials

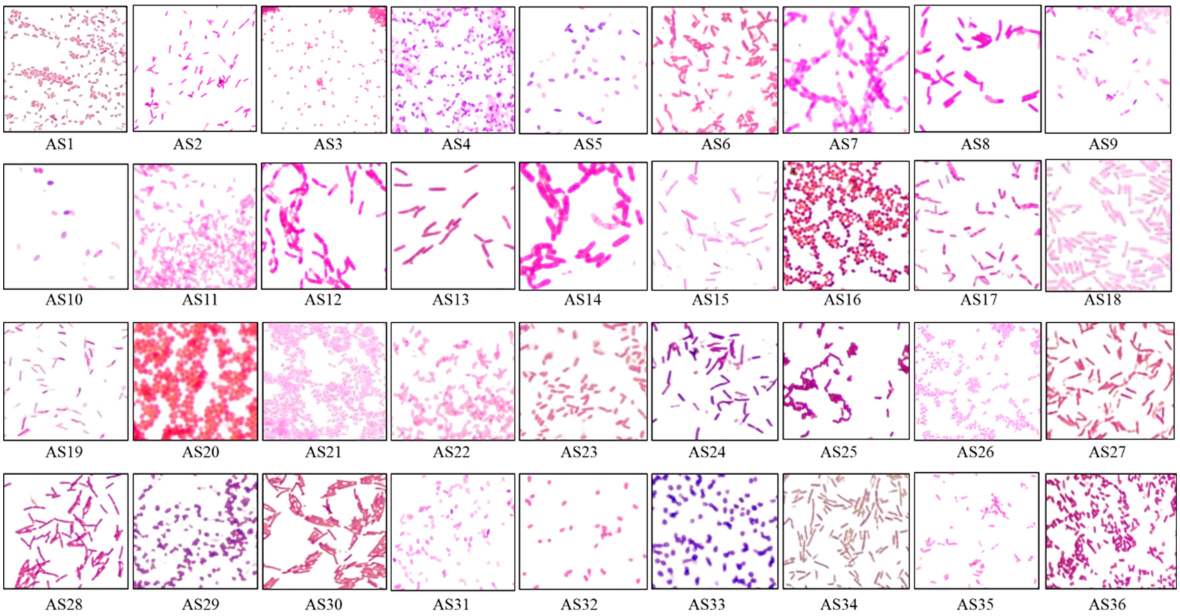

Figure S1. Microscopic analysis of isolated microbial strains.

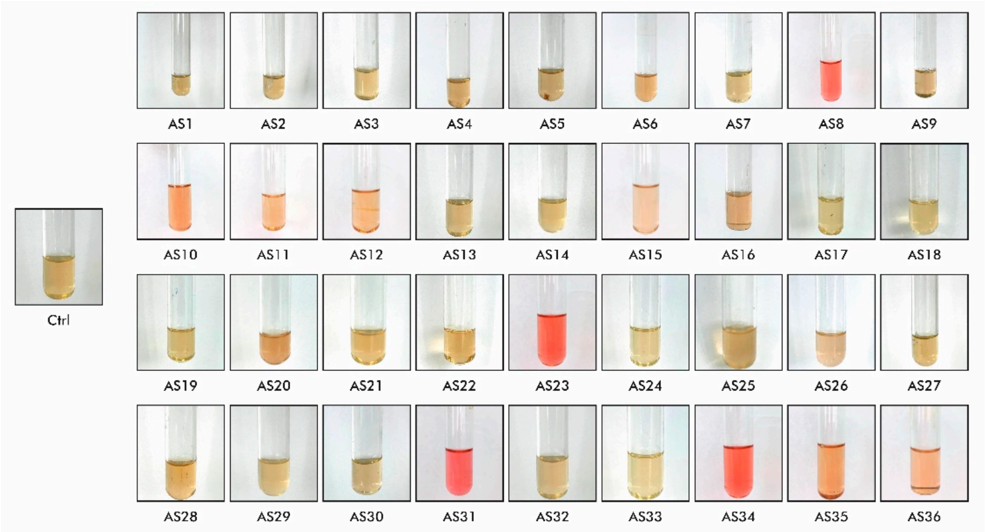

Figure S2. Qualitative analysis of IAA production by isolated microbial strains.

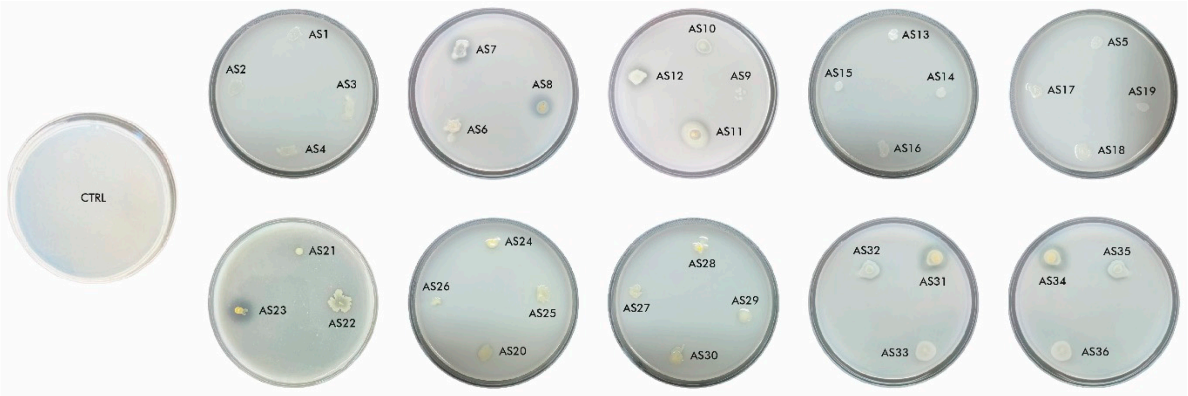

Figure S3. Qualitative analysis of phosphates solubilisation by isolated microbial strains.

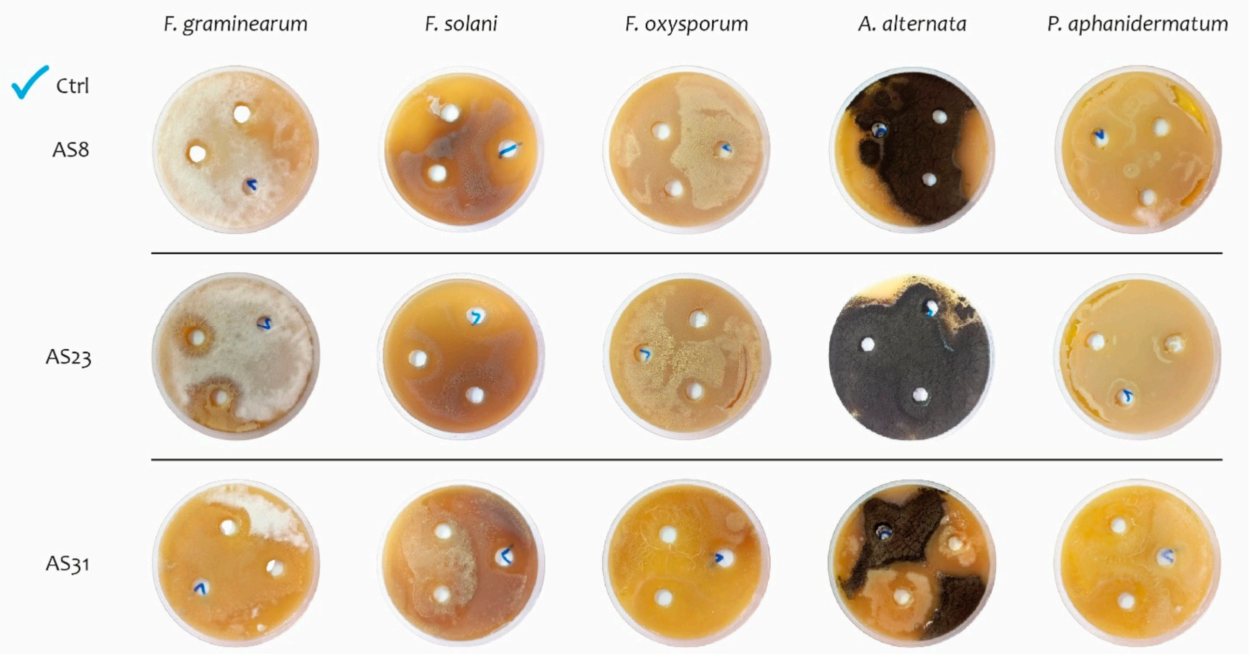

Figure S4. Antifungal activity of selected isolates.

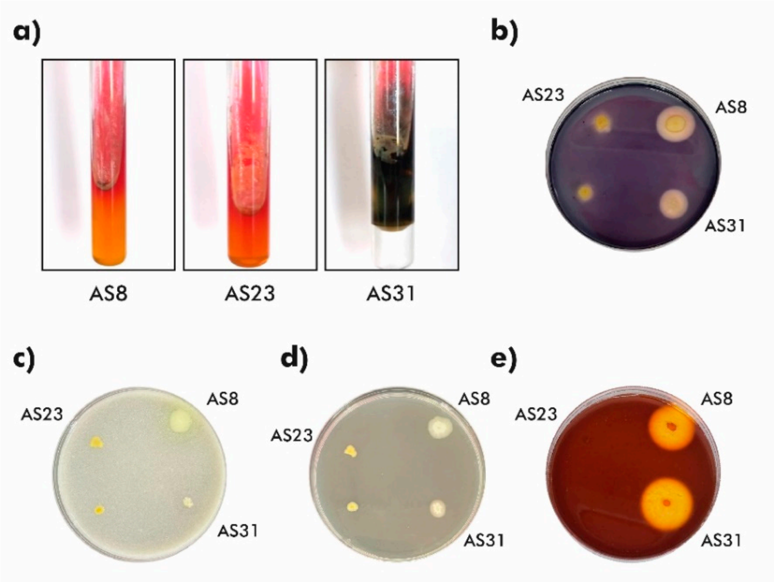

Figure S5. Biochemical properties of selected isolates: a) carbohydrates fermentation; b) amylolytic activity; c) protease activity; d) lipase activity; e) cellulolytic activity.

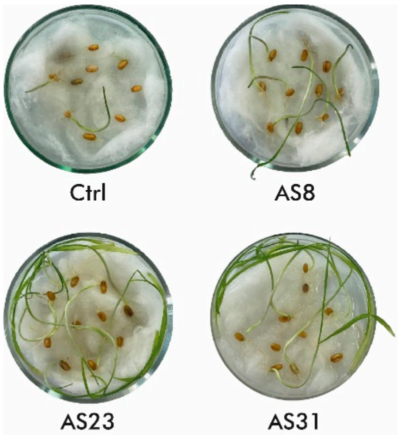

Figure S6. Influence of selected isolates on *Triticum aestivum* seed germination.
